# Dietary fibre and the gut microbiome: implications for glucose homeostasis

**DOI:** 10.1097/MCO.0000000000001160

**Published:** 2025-08-26

**Authors:** Jennifer E. Pugh, Edward S. Chambers

**Affiliations:** Section of Nutrition, Department of Metabolism, Digestion and Reproduction, Faculty of Medicine, Imperial College London, London, UK

**Keywords:** dietary fibre, glycaemic control, gut barrier integrity, gut microbiota, short-chain fatty acids

## Abstract

**Purpose of review:**

Despite long-standing evidence linking dietary fibre intake to improved glycaemic control and reduced chronic disease risk, most individuals fail to meet recommended intake levels. As interest grows in the gut microbiota's role in mediating fibre's health effects, this review evaluates recent human intervention trials to assess whether dietary fibre improves glucose homeostasis through microbiota-dependent mechanisms.

**Recent findings:**

Nine recent randomised controlled trials (RCTs) have examined the effects of dietary fibre on glycaemic markers and gut microbiota, primarily in individuals at risk of or diagnosed with metabolic disease. Five studies reported improvements in glycaemic outcomes such as fasting glucose, insulin, or HOMA-IR. Microbial responses were inconsistent, with variable effects on diversity and composition. Notably, improvements in markers of gut barrier integrity and systemic inflammation were consistently observed in studies including these as mechanistic outcomes.

**Summary:**

Although dietary fibre may enhance glycaemic control and modulate the gut microbiota, effects vary by fibre type, dose, population, and study design. Markers of gut barrier integrity and inflammation appear to be more reliable indicators of benefit compared with the assessments of gut microbial composition. Future trials should prioritise healthy populations to explore the potential of dietary fibre to maintain metabolic health.

## INTRODUCTION

Prospective cohort studies consistently demonstrate that high dietary fibre intake, defined as the consumption of carbohydrate polymers that resist digestion and absorption in the small intestine, is associated with a reduced risk of noncommunicable metabolic diseases, common cancers, and all-cause mortality [1]. High fibre intake has also been linked to lower blood glucose levels, a shared risk factor for many chronic health conditions [2]. Although the health benefits of dietary fibre are well established, particularly its role in supporting glucose homeostasis, an estimated 90% of adults worldwide do not meet the recommended daily intake [3]. Increasing population-level fibre intake therefore represents a widely applicable and cost-effective strategy to improve metabolic health and reduce incidence of common chronic diseases.

Although all dietary fibres contribute towards meeting recommended intake levels, individual fibres differ in their physiochemical properties and, consequently, in the health benefits they confer. One established mechanism by which dietary fibre improves glucose homeostasis involves the action of viscous soluble fibres, such as β-glucan, guar gum, and pectins, which can attenuate postprandial glucose responses by slowing gastric emptying and nutrient absorption [4]. Beyond these direct gastrointestinal effects, dietary fibre has a profound influence on the gut microbiome. Specific fibres, particularly fermentable soluble fibres such as inulin and resistant starch, are metabolised by gut bacteria, leading to shifts in microbial composition and metabolic activity [5]. Increased fibre intake has been associated with elevated production of short-chain fatty acids (SCFAs) and reduced levels of microbial metabolites linked to proteolytic fermentation and secondary bile acid production [5]. Microbial-derived metabolites exert both local and systemic effects. For instance, SCFAs play a crucial role in maintaining intestinal health by stimulating mucosal secretion and promoting the expression of tight-junction proteins, thereby strengthening gut barrier integrity [6]. Once absorbed into the circulation, SCFAs also modulate host metabolism by enhancing insulin secretion from pancreatic β-cells [7], reducing lipolysis in adipose tissue [7], and increasing glucose uptake in skeletal muscle [8]. Together, these physiological actions would support glucose homeostasis and illustrate how high-fibre diets may confer glycaemic benefits through microbiota-driven metabolic changes. 

**Box 1:**
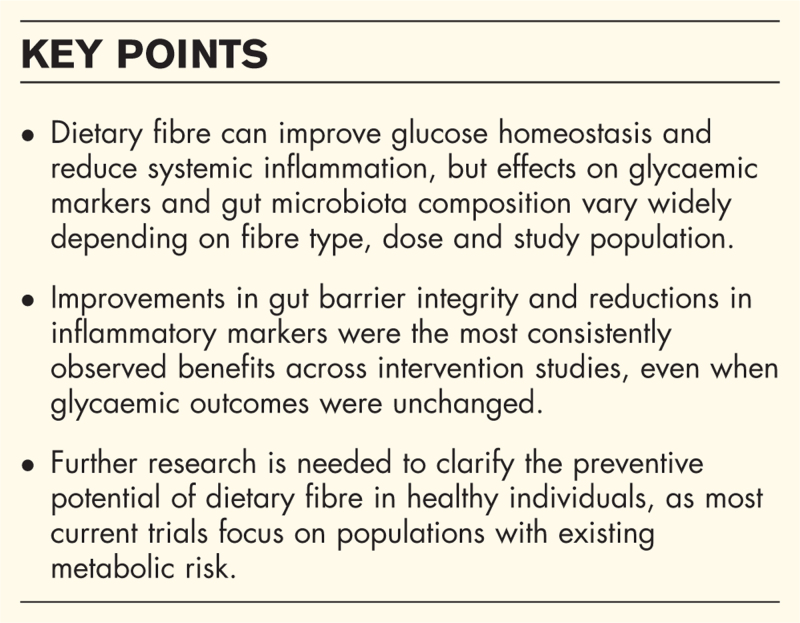
no caption available

## AIM OF THE REVIEW

This review aims to evaluate recent human intervention trials investigating the impact of increased dietary fibre intake on markers of glucose homeostasis. Special emphasis is placed on studies that assess changes in gut microbial composition and/or metabolic activity, with the goal of identifying shared mechanistic pathways through which dietary fibre influences glucose regulation. By elucidating these mechanisms, the review seeks to highlight potential novel therapeutic targets that could be leveraged to enhance metabolic health through dietary or microbiota-directed interventions.

## DIETARY FIBRE INTERVENTIONS, GLYCAEMIA, AND THE GUT MICROBIOTA

The literature search identified nine randomised controlled trials (RCTs) investigating the effects of dietary fibre interventions on glycaemia and gut microbial outcomes across various populations in the last 18 months (since September 2023).

These studies (Table 1, Table 1, Supplemental Digital Content) mostly recruited individuals with preexisting conditions, including metabolic dysfunction-associated steatotic liver disease (MASLD) (three studies), prediabetes (one study), excess body weight (two studies), Helicobacter pylori (HP) infection (one study), and those at risk of cardiovascular disease (CVD) (one study), as well as healthy volunteers (one study). Study sample size ranged from 19 to 200 participants and study durations spanning from 14 to 120 days.

**Table 1:** Study design and participant characteristics

Study	Study population	Sample size (*n*)	Age (years) Mean ± SD	BMI (kg/m^2^) Mean ± SD	Design & duration	Intervention	Control	Outcomes measured	Factors affecting glycaemic response	Notes
[12]	HV	32 (18 M, 14 F)	31.3 ± 10.1	25.7 ± 4.8	RCT, parallel, 14 days	4.8 g fibre croissant^a^	1.3 g fibre croissant	Body composition Biochemistry Dietary intake Subjective appetite Polyphenols Gut microbiota	No change to habitual diet, no change to physical activity	No diet/activity change
[9]	Prediabetes	50 (23 M, 27 F)	52.3 ± 9.9	30.7 ± 5.5	RCT, parallel, 84 days	5 g GOS, EPS & konjac glucomannan	5 g cellulose	Body composition Biochemistry Inflammatory markers Gut microbiota Dietary intake	10 hr fast 12 hrs abstain from exercise 24hr abstain from alcohol	Gender not balanced between groups Sig differences in baseline characteristics and dietary intake
[14^▪▪^]	At risk of CVD	30 (15 M, 15 F)	44.1 ± 8.2	28.8 ± 2.2	RCT, crossover, 56 days 6- to 8-week washout	18 g cranberry fibre biscuit	Low-fibre biscuit	Body composition Biochemistry endotoxemia Inflammation Gut microbiota Metabolomics Dietary intake	8 hr fast Low dietary fibre meal	8 h fast; low fibre meal
[15]	MASLD	26	NR	29.1 ± 3.4	RCT, parallel, 60 days	High fibre rolls^a^ (24 g fibre)	Low fibre rolls (12 g fibre)	Body composition Biochemistry Gut microbiota SCFA		Dietitian guidance
[10]	Individuals with HP infection	158	44.9 ± 13.2	24.2 ± 4.0	RCT, parallel, 84 days	Fermented rye bran	Refined wheat products	Biochemistry Body composition Gut microbiota Metabolomics SCFA/bile acids		Secondary analysis 10% reduction in fasting glucose = 36% cohort Fibre dose not reported
[11^▪▪^]	Excess body weight	37 (22 M, 15 F)	33.43 ± 7.71	28.7 ± 3.8	RCT, crossover, 56 days, 4-week washout	40 g HAM-RS2	~18 g corn starch	Body composition Biochemistry Gut microbiota SCFA Metabolomics Dietary intake	10-h fast substantial weight loss	meals provided Energy-matched control, exact weight not reported
[16]	Overweight young adults	59 (40 M, 19 F)	28.3 ± 6.55	27.3 ± 1.4	RCT, crossover 14 days, 14-day washout	30 g inulin	16 g maltodextrin	Body composition Biochemistry Gut microbiota SCFA Metabolomics Dietary intake	Average 12.5-h fast	Energy matched control
[13^▪▪^]	MASLD	200 (145 M, 55 F)	39.1 ± 9.1	28.5 ± 0.4	RCT, parallel, 120 days	40 g HAM-RS2	40 g corn starch	Body composition Biochemistry Taxonomic profile Functional profiling Metabolomics Dietary intake	Substantial weight loss	Dietary counselling provided
[17]	MASLD	19 (15 M, 4 F)	50 ± 11	32.5 ± 1.1	RCT, parallel, 84 days	16 g inulin and oligofructose	16 g maltodextrin	Body composition Biochemistry Gut microbiota		Weight maintenance counselling

a Fibre from a mix of sources.

CVD, cardiovascular disease; EPS, exopolysaccharides; F, female; GOS, galacto-oligosaccharides; HAM-RS2, high-amylose maize-resistant starch type 2; HV, healthy volunteers; M, male; MASLD, metabolic dysfunction-associated steatotic liver disease; NR, not reported; RCT, randomised controlled trial; SCFA, short-chain fatty acids.

Fibre interventions were highly varied, including supplements consisting of resistant starch, cranberry fibre, fermented rye bran, inulin and oligofructose, galacto-oligosaccharides (GOS), exopolysaccharides (EPS) and konjac glucomannan as well as high-fibre baked goods (biscuits, rolls and croissants).

Some studies attempted to control factors influencing glycaemic responses by asking participants to fast and abstain from physical activity prior to study visits. Some monitored dietary intake throughout the duration of the study. In one case, dietary counselling was provided to ensure weight was maintained. In another, meals were provided for the study duration. However, as glucose control was not often the primary outcome for many of the studies, several studies reported substantial weight loss or metabolic improvements. Limitations of the studies included imbalances in gender distribution between control and intervention groups and not reporting fibre doses. The different fibre types and populations recruited for these studies underscore the heterogeneity in study designs and interventions, leading to the disparity in results seen for this review.

### Studies reporting improvements in glucose homeostasis with dietary fibre intervention

Beteri *et al.* [9] examined a blend of galacto-oligosaccharides (GOS), exopolysaccharides (EPS), and konjac glucomannan in individuals with prediabetes. Over 84 days, they observed reductions in HbA1c, improvements in microbial alpha-diversity, and lower levels of lipopolysaccharide-binding protein (LBP), a marker of gut barrier integrity. However, an imbalance in gender distribution between the two groups resulted in physiologically meaningful differences in baseline characteristics and dietary intake between groups, which may have influenced the study results. Similarly, Li *et al.* [10] conducted two distinct studies exploring the metabolic effects of fibre. One focused on fermented rye bran in HP-infected individuals, finding that responders, those with ≥10% reductions in fasting glucose, showed distinct microbiota profiles and increased serum butyrate and bile acids, despite no change in faecal SCFA concentrations. In a separate trial, Li *et al.* [11^▪▪^] reported that supplementation with high-amylose maize resistant starch type 2 (HAM-RS2) in overweight adults improved postprandial glucose and insulin responses, reduced inflammatory cytokines (TNF-α and IL-1β), and altered microbiota composition, most notably increasing *Bifidobacterium* species alongside a decreased concentration of products of protein fermentation (isobutyrate and valerate) in stool samples.

The impact of dietary fibre intervention on glucose metabolism was also supported in a short-term trial by Barone Lumaga *et al.* [12], who demonstrated that a high-fibre croissant containing both soluble and insoluble fibres reduced fasting blood glucose over 14 days. Microbial shifts were noted (reductions in populations of *Faecalicatena fissicatena* and *Faecalibacterium prausnitzii* and an increase in *Candidatus Cibiobacter qucibialis*), though faecal metabolites were not assessed. More robust effects were seen in a longer trial by Ni *et al.* [13^▪▪^], who recruited individuals with MASLD for a 120-day HAM-RS2 intervention. This study revealed significant improvements in insulin sensitivity (lower fasting and postprandial insulin, C-peptide, and HOMA-IR), reductions in TNF-α, and a decline in circulating LPS, suggesting enhanced gut barrier integrity. Interestingly, while microbial diversity decreased, certain taxa such as *Clostridium nexile* and *Ruminococcus bromii* increased.

### Studies reporting no evidence of improvements in glucose homeostasis with dietary fibre intervention

Not all RCTs found evidence of improvements in glycaemic outcomes. Hornero-Ramirez *et al.* [14^▪▪^] found no changes in glycaemic response following a biscuit-based fibre intervention in individuals at risk of cardiovascular disease (CVD), though shifts in microbiota composition (e.g., increased *Bacteroides* spp.) were observed. Furthermore, the investigation reported significantly lower levels of faecal calprotectin, an established marker of gut inflammation, following the fibre intervention. Kaźmierczak-Siedlecka *et al.* [15] similarly reported no glycaemic improvements in MASLD patients consuming high-fibre rolls, despite enhanced microbial diversity and increased acetate and butyrate in stool. In shorter interventions, Medawar *et al.* [16] and Reshef *et al.* [17] both used inulin-based prebiotics but found no significant changes in glucose metabolism or inflammatory markers, even though both reported the expected bifidogenic effects and compositional shifts in the gut microbiota. Notably, Medawar *et al.* observed decreased microbial diversity, while Reshef *et al.* recorded a fourfold increase in *Bifidobacterium* spp., reinforcing the variability in metabolic outcomes despite consistent microbiota compositional changes.

## DISCUSSION

Across the nine recently published RCTs investigating the effects of fibre supplementation on the gut microbiota and glycaemic response, few consistent patterns emerge. This variability in results can be partially attributed to heterogeneity in fibre type and dosage, participant characteristics or disease status, and study duration. Furthermore, whilst the growing body of evidence supporting the benefits of dietary fibre in metabolic conditions like MASLD is encouraging, the lack of consensus on gut microbiota and glycaemic control in healthy individuals warrants further investigation. Given the potential for dietary fibre to prevent metabolic disease [1], greater emphasis should be placed on exploring its role in maintaining good glycaemic control in healthy populations. This is particularly important given that the global economic cost of metabolic diseases such as type 2 diabetes is estimated to exceed $1.3 trillion annually [18], highlighting the urgent need for cost-effective, population-level prevention strategies.

Improvements in glycaemic markers, including reductions in fasting glucose, fasting and postprandial insulin and HOMA-IR were observed in three studies which supplemented resistant starch or a fibre combination [9,11^▪▪^,^13^^▪▪^], whereas, some studies reported no significant differences in glycaemia between the intervention and control [10,14^▪▪^,^15^,^16^]. These null results may potentially stem from differences in fibre fermentability, insufficient fibre dose or intervention length.

Although a few studies reported increases in beneficial bacterial taxa, most notably *Bifidobacterium* [11^▪▪^,^16^,^17^], the effects of dietary fibre supplementation on gut microbial composition were highly variable across RCTs. Changes to specific taxa were inconsistent, and shifts in microbial diversity also varied: some studies reported a reduction in alpha diversity [13^▪▪^,^16^] while others observed increased diversity [9,15]. A decline in microbial diversity is not unexpected when a single fibre type is supplemented at a high dose, as it tends to selectively enrich bacteria capable of utilising that specific substrate, thereby reducing overall community diversity.

In studies assessing gut microbial metabolites in stool and serum, increases in saccharolytic fermentation products (SCFAs) and reductions in proteolytic fermentation by-products (BCFAs) were generally observed, aligning with expected outcomes. However, these findings were not consistent across all RCTs, likely due to variations in fibre type, dose, and intervention duration. Notably, studies that measured markers of gut barrier integrity, such as LPS, LBP, and faecal calprotectin, consistently reported improvements following dietary fibre intervention. Given the well established link between gut barrier function, systemic inflammation, and glucose homeostasis, these findings are significant. Several studies also reported reductions in inflammatory cytokines, further supporting the role of dietary fibre in modulating inflammation. These observations highlight the importance of including markers of gut barrier integrity and systemic inflammation in future dietary fibre intervention studies to better understand the contribution of this mechanism to metabolic health improvements and maintenance.

## CONCLUSION

In summary, recent human intervention studies support the potential of dietary fibre to improve glycaemic markers such as fasting glucose, insulin, and HOMA-IR, although not all studies demonstrated significant metabolic effects. This inconsistency likely stems from variations in fibre type, dosage, intervention duration, and participant characteristics. Similarly, changes in gut microbiota composition were heterogeneous, with inconsistent effects on microbial diversity and specific taxa. Notably, improvements in markers of gut barrier integrity and systemic inflammation were consistently reported, indicating these may serve as more robust mechanistic indicators of the benefits of dietary fibre. As most trials to date have focused on individuals with existing metabolic risk, further research is needed to clarify whether dietary fibre can offer long-term preventive effects in metabolically healthy populations.

## FUTURE DIRECTIONS

To advance our understanding of dietary fibre's role in metabolic health, future research should focus on the following areas:

(1) *Standardisation of fibre supplementation trials*
  Variability in fibre type, dosage, study duration, and selected mechanistic endpoints has hindered cross-study comparison. The adoption of standardised protocols, including consistent microbial sequencing methods, stool metabolite measurements, and glycaemic outcome assessments, would enhance support the identification of fibre interventions that reliably improve or maintain metabolic health.
(2) *Longitudinal trials in healthy populations*
  Most current RCTs individuals with existing metabolic dysfunction. To determine whether fibre supplementation can prevent glycaemic dysregulation and other early markers of metabolic disease, well powered, long-term trials in metabolically healthy individuals are warranted, particularly given aforementioned results from observational studies.
(3) *Focus on gut barrier integrity and inflammatory markers*
  Improvements in gut barrier integrity and inflammatory markers were among the most consistently observed outcomes following dietary fibre supplementation. These mechanistic endpoints should be prioritised in future trials to better clarify the role of fibre in promoting metabolic health.

## Acknowledgements

*None.*

### Financial support and sponsorship

*None.*

### Conflicts of interest

*There are no conflicts of interest.*

## Supplementary Material

Supplemental Digital Content

## REFERENCES AND RECOMMENDED READING

Papers of particular interest, published within the annual period of review, have been highlighted as:

▪ of special interest
▪▪ of outstanding interest
