# Supplementary material for: Dietary fibre and the gut microbiome: implications for glucose homeostasis
Source: Curr Opin Clin Nutr Metab Care. 2025 Aug 26;28(6):483–8. doi: 10.1097/MCO.0000000000001160 (PMC12517706; doi:10.1097/MCO.0000000000001160)
Supplement: Supplemental Digital Content [file cocnm-28-483-s001.docx]

**Supplementary Table 1:** Summary of notable study results

| **Study** | **Glycaemic / Metabolic Outcomes** | **Inflammatory marker Outcomes** | **Microbiota Outcomes** | **Bacterial metabolite Outcomes** |
| --- | --- | --- | --- | --- |
| [Barone Lumaga, 2024](https://www.sciencedirect.com/science/article/pii/S0022316624004668?via%3Dihub) | ↓ fasting blood glucose  ↔ fasting insulin  ↔ HOMA-IR | ↔ CRP | ↓ Faecalicatena fissicatena, Faecalibacterium prausnitzii  ↑ Candidatus Cibiobacter qucibialis | Not measured |
| [Beteri, 2024](https://www.mdpi.com/2072-6643/16/14/2205) | ↓ HbA1c | ↓ LBP (↓ LPS) | ↑ alpha-diversity | ↓ LBP (↓ LPS) |
| [Hornero-Ramirez, 2024](https://www.tandfonline.com/doi/full/10.1080/19490976.2024.2438823?rfr_dat=cr_pub++0pubmed&url_ver=Z39.88-2003&rfr_id=ori%3Arid%3Acrossref.org#abstract) | ↔ glycaemic response | ↓ faecal calprotectin | ↑ Bacteroides ovatus, B. uniformis, Agathobaculum butyriciproducens | ↓ valine, isoleucine and glutamate |
| [Kaźmierczak-Siedlecka, 2024](https://www.mdpi.com/2072-6643/16/8/1173) | ↔ glycaemic response | Not measured | ↑ phylum-level diversity | ↑ faecal butyrate and acetate increased within intervention group |
| [Li, 2024a](https://onlinelibrary.wiley.com/doi/full/10.1002/mnfr.202400274?saml_referrer) | ↔ fasting glucose overall, ↓ 10% fasting glucose in 36% participants | ↔ CRP | Four CAGs identified to be different between responder and non-responder groups: Bacteroides (CAG1) was greater | ↔ faecal SCFA  ↑ serum butyrate  ↑ primary bile acids  ↓ secondary bile acids |
| [Li, 2024b](https://www.nature.com/articles/s42255-024-00988-y) | ↓ body weight  ↓ glucose AUC  ↓ postprandial insulin | ↓ TNF-α  ↓ IL-1β | ↑ B. adolescentis, B. longum  ↓ R. bromii, A. putredinis, B. vulgatus, O. splanchnicus, P. merdae | ↓ isobutyrate and valerate  ↔ acetate, propionate, butyrate, hexanoate |
| [Medawar, 2024](https://gut.bmj.com/content/73/2/298.long) | ↔ glycaemic response | ↔TNF-α  ↔IL-6 | ↓ richness and alpha diversity after prebiotics  ↑ *Anaerostipes, Bifidobacterium, Collinsella, Holdemanella, Lacticaseibacillus, Lactiplantibacillus,*  *Lactobacillus*, Ligilactobacillus, Limosilactobacillus*  ↓ *Desulfovibrio, Eggerthella, Roseburia, Shuttleworthia, Subdoligranulum* | ↔ SCFA |
| [Ni, 2023](https://www.cell.com/cell-metabolism/fulltext/S1550-4131(23)00297-8?_returnURL=https%3A%2F%2Flinkinghub.elsevier.com%2Fretrieve%2Fpii%2FS1550413123002978%3Fshowall%3Dtrue) | ↓ fasting and postprandial insulin  ↓ fasting C-peptide  ↓ HOMA-IR  ↔ fasting and postprandial glucose | ↓ TNF-α  ↓ LPS | ↓ alpha- and beta-diversity  ↑ Clostridium nexile, Streptococcus salivarius, Ruminococcus bromii, Prevotella copri, Megasphaera (unclassified) | ↔ faecal SCFA |
| [Reshef, 2024](https://www.mdpi.com/2072-6643/16/11/1571) | ↔ glycaemic response | ↔ CRP | ↑ Bifidobacterium (4-fold) | Not measured |
| AUC, Area Under the Curve; CAG, Co-abundance Group; CRP, C-reactive protein; DMM, Dirichlet Multinomial Mixtures; HbA1c, Hemoglobin A1c; HOMA-IR, Homeostatic Model Assessment of Insulin Resistance; IL-1β, Interleukin-1 beta; IL-6, Interleukin-6; LBP, Lipopolysaccharide Binding Protein; LPS, Lipopolysaccharide; SCFA, Short-chain Fatty Acids; TNF-α, Tumor Necrosis Factor-alpha | | | | |
